# Association between NSAIDs and *Clostridium difficile*-Associated Diarrhea: A Systematic Review and Meta-Analysis

**DOI:** 10.1155/2016/7431838

**Published:** 2016-03-08

**Authors:** Nitipong Permpalung, Sikarin Upala, Anawin Sanguankeo, Suthanya Sornprom

**Affiliations:** ^1^Division of Infectious Diseases, Beth Israel Deaconess Medical Center, Harvard Medical School, Boston, MA 02115, USA; ^2^Department of Internal Medicine, Bassett Medical Center and Columbia University College of Physicians and Surgeons, Cooperstown, NY 13326, USA; ^3^Department of Preventive and Social Medicine, Faculty of Medicine Siriraj Hospital, Mahidol University, Bangkok 10700, Thailand

## Abstract

*Objective. Clostridium difficile* infection is a leading cause of nosocomial diarrhea in developed countries. Studies evaluating the associations of increased risk of community-acquired CDAD and the use of nonsteroidal anti-inflammatory drugs (NSAIDs) have yielded inconclusive results. We conducted a systematic review and meta-analysis to compare the odds of NSAID exposure in patients with CDAD versus patients without CDAD in both community-based and healthcare-associated settings.* Methods.* Relevant observational studies indexed in PubMed/MEDLINE and EMBASE up to February 2015 were analyzed and data were extracted from nine studies. Of these, eight studies were included in the meta-analysis.* Results.* A search of the databases resulted in 987 articles. The nine studies from which data were extracted involved over 39,000 subjects. The pooled odds ratio for history of NSAID use in participants with CDAD compared with controls was 1.41 (95% CI 1.06–1.87; *p* < 0.01), indicating a significant increased odds of CDAD among patients exposed to NSAIDs.* Conclusions.* To the best of our knowledge, this is the first study of its nature to demonstrate the association between the use of NSAIDs and increased risk of CDAD. Further studies to evaluate if any specific types of NSAIDs can increase the risk of CDAD are warranted.

## 1. Introduction

*Clostridium difficile* (*C. difficile*) infection is the leading cause of* C. difficile*-associated diarrhea (CDAD), an important type of nosocomial diarrhea in developed countries [1]. The disease spectrum of CDAD could range from asymptomatic colonization to fulminant colitis. The incidence of CDAD cases has increased exponentially over the past decade, and a higher proportion of cases were reported as community-acquired [2, 3]. The classic pathogenesis of CDAD was attributed to antimicrobial use with the consequent alteration of the intestinal microflora, thereby causing* C. difficile* overgrowth. However, the use of acid-suppressive therapy, including proton pump inhibitors and H_2_-receptor antagonists, was also associated with an increased risk for community-acquired CDAD [4, 5]. The elevated pH in gastric acid might enhance* C. difficile* survival. Further, PPI use, irrespective of treatment length, could alter gene expression in human colonic cell lines, resulting in decreased colonocyte integrity [6, 7]. A possible association was reported between nonsteroidal anti-inflammatory drugs (NSAIDs), especially diclofenac, and community-acquired CDAD in patients that were not recently hospitalized or exposed to antimicrobial agents [8, 9]. Dial et al. conducted a population-based case-control study to evaluate the association between the use of acid-suppressive agents and the risk of CDAD [5]. Interestingly, they found an unexpected association between the use of NSAIDs and an increased risk of CDAD. Subsequent studies were conducted to evaluate this association; however, the results were subject to debate [10–16]. Thus, to investigate this association further, we performed a systematic review and meta-analysis of observational studies to compare the odds of NSAID exposure in patients with CDAD versus patients without CDAD in both community-based and healthcare-associated settings.

## 2. Materials and Methods

This systematic review and meta-analysis was conducted and reported according to the established guideline for meta-analysis [17] and was registered in PROSPERO (registration number: CRD42014014671).

### 2.1. Types of Studies

All published and unpublished randomized controlled trials and observational studies including prospective cohort, retrospective cohort, case-control, and cross-sectional studies, involving patients infected with* Clostridium difficile*, were included. Reviews, case reports, letters, and commentaries were not included.

### 2.2. Types of Outcome Measures

The primary outcome was the comparison of the number of participants with a history of NSAID exposure and CDAD versus those without CDAD.

### 2.3. Search Methods for Identification of Studies

Anawin Sanguankeo and Sikarin Upala independently searched published studies indexed in the PubMed/MEDLINE and EMBASE from database inception to October 2014. References of selected retrieved articles were also examined. The search terms used were* Clostridium difficile*, pseudomembranous colitis, hospital acquired diarrhea, antibiotic-associated diarrhea, NSAID, and common generic NSAIDs. Further details of the strategy used for the literature search are included in Supplemental Data, in Supplementary Material available online at http://dx.doi.org/10.1155/2016/7431838.

### 2.4. Data Collection and Analysis

#### 2.4.1. Selection of Studies

Anawin Sanguankeo and Sikarin Upala independently reviewed the titles and abstracts of all citations that were identified. After all the studies were abstracted, face-to-face data comparisons were conducted between investigators to ensure completeness and reliability. The inclusion criteria were independently applied to all identified studies. Differing decisions were resolved by consensus.

#### 2.4.2. Data Extraction and Management

Full-text versions of potentially relevant papers identified in the initial screening were retrieved. If multiple articles from the same study were found, only the article with the longest follow-up period was included. Data concerning study design (cross-sectional, case-control, prospective cohort, and retrospective cohort), participant characteristics (age, sex, and settings), NSAID use (previous or current use, overall, and specific NSAID use), and outcome measures (definition of CDAD, number of participants, odds ratio, or risk ratio) were independently extracted. We contacted the authors of the primary reports to request any unpublished data. If the authors did not reply, we used the available data for our analyses.

### 2.5. Assessment of Bias Risk

The quality of observational studies (OBS) was evaluated by each investigator using the Newcastle-Ottawa quality assessment scale [19].

### 2.6. Statistical Methods

Data analysis was performed using the Comprehensive Meta-Analysis 3.3 software from the Biostat, Inc. We reported the estimated pooled odds ratio (OR) of NSAID use using a random effects model because of the high likelihood of heterogeneity among the studies. Subgroup analyses were performed based on types of NSAID, duration of NSAID (60 days or less and more than 60 days), age (50 years or less and more than 50 years), and risk of bias (high and low risk of bias). The heterogeneity of the effect size estimates across these studies was quantified using the *I* ^2^ and *Q* statistics [20]. Possible publication bias was assessed using funnel plot and Egger's regression test [21]. Meta-regression was not performed because there were not enough studies for this analysis.

## 3. Results

### 3.1. Description of Included Studies

**Figure 1:**
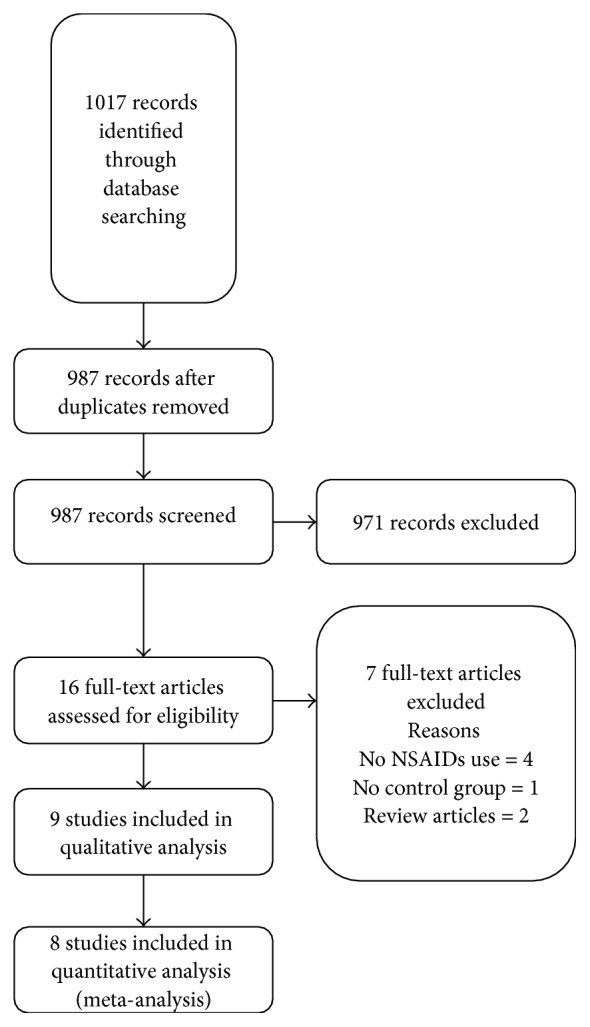
Results of information search.

The initial search yielded 987 articles (Figure 1); 971 articles were excluded because they were letters or review articles, the participants did not have CDAD, or there were no records of use of NSAID.

A total of 16 articles underwent full-length review. Finally, data were extracted from nine observational studies [5, 10–16, 18] involving 39,309 participants. Eight of these studies that reported outcomes of interest (number of participants with a history of NSAID exposure and CDAD versus those without CDAD) were included in the meta-analysis. The characteristics of the extracted studies are outlined in Table 1.

### 3.2. Risk of Bias and Quality Assessment

Quality assessment scores using the Newcastle-Ottawa Scale tool for observational studies are summarized in Table 2. Most studies had a score of 3-4 in the selection scale, a score of 2 in the comparability scale, and score of 2 in the exposure scale. All studies applied either interview questionnaires, self-reports, or medical records to assess exposure. Two studies did not describe control methods for both study groups regarding study design or analysis [12, 16].

### 3.3. Quantitative Results (Meta-Analysis)

**Figure 2:**
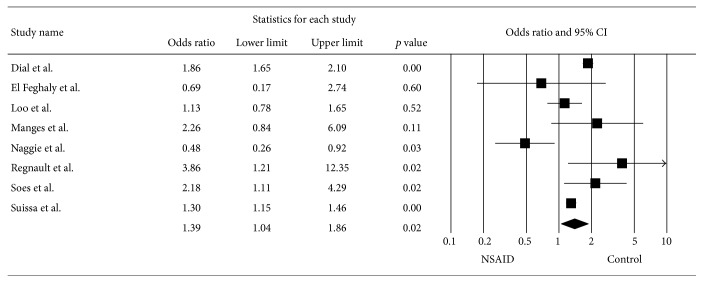
Forest plot of the included studies comparing odds ratio of CDAD in patients who used NSAID and those who did not.

**Figure 3:**
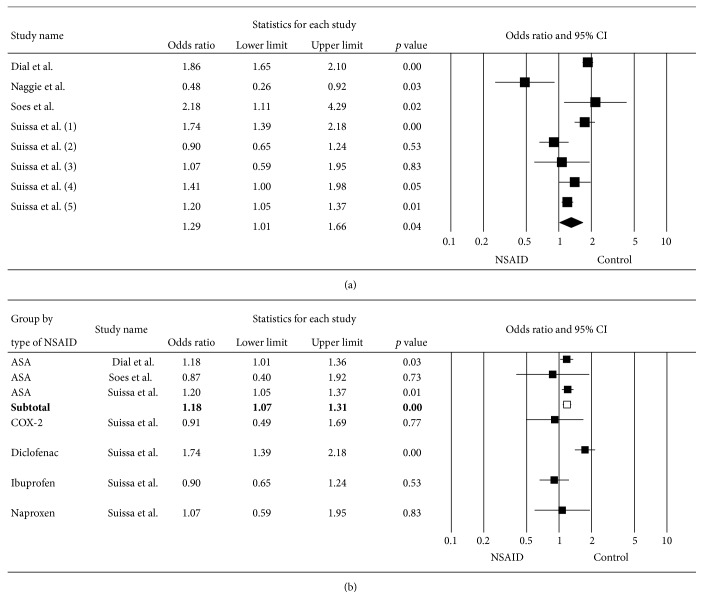
Forest plot of subgroup analysis in (a) nonselective NSAID and (b) each type of NSAID.

**Figure 4:**
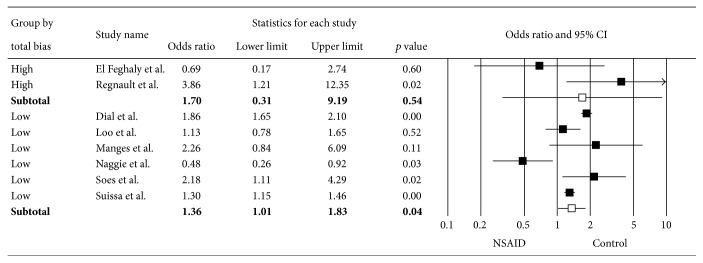
Forest plot of subgroup analysis by risk of bias.

**Figure 5:**
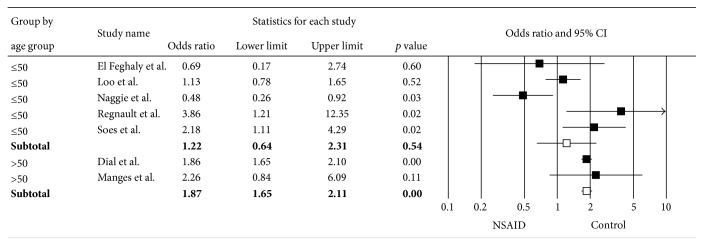
Forest plot of subgroup analysis by age group.

**Figure 6:**
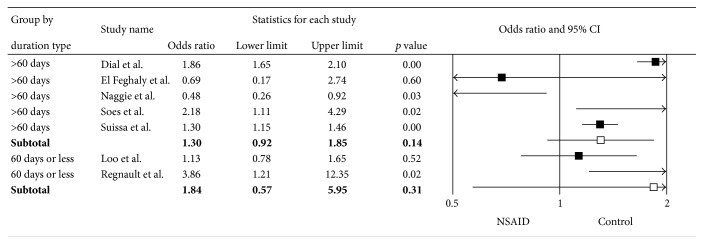
Forest plot of subgroup analysis by duration of NSAID use.

The meta-analysis was performed using the random effects model (Figure 2). It revealed that the pooled OR for NSAID use in participants with CDAD compared with controls was 1.39 (95% CI 1.04–1.86; *p* = 0.02). The statistical heterogeneity among the studies was moderate to high, with an *I* ^2^ of 89%. Subgroup analyses based on the NSAID type (Figures 3(a) and 3(b)), risk of bias (Figure 4), age (Figure 5), and duration of NSAID use (Figure 6) were shown. Nonselective NSAID (excluding COX-2 inhibitors) was significantly associated with CDAD with pooled OR = 1.29 (95% CI 1.01–1.66; *p* = 0.04). The pooled OR of studies with low risk of bias was 1.36 (95% CI 1.01–1.83; *p* = 0.04), while studies with high risk of bias had pooled OR = 1.70 (95% CI 0.31–9.19; *p* = 0.54). Studies with mean age of 50 years and older had OR = 1.87 (95% CI 1.65–2.11; *p* < 0.01), while those with mean age less than 50 years had OR = 1.22 (95% CI 0.64–2.31; *p* = 0.54). Subgroup analysis of duration of NSAID use did not show a significant difference of CDAD in both shorter (OR = 1.84) and longer (OR = 1.30) duration.

### 3.4. Sensitivity Analysis

Sensitivity analysis was performed using a fixed effects model rather than a random effects model. The result of the point estimate and its statistical significance were not different from the main result.

### 3.5. Publication Bias

To investigate potential publication bias, we examined the contour-enhanced funnel plot of the included studies. Vertical axis represents study size (standard error) while horizontal axis represents effect size (log odds ratio). From this plot, bias is not present because there is symmetrical distribution of studies on both sides of the mean. Egger's regression test for bias was −0.45 (95% CI, −3.50 to 2.60) (Supplemental Figure 1).

## 4. Discussion

To the best of our knowledge, this is the first systematic review and meta-analysis of published observational studies to demonstrate the association between the use of NSAIDs and CDAD. The results showed that odds of CDAD among patients with NSAID exposure were significantly increased. This association between NSAID and CDAD was found in nonselective NSAID and patients who were 50 years or older, regardless of duration of NSAID use.

It should be noted that the case definitions of CDAD in the individual studies were not exactly the same. The first prescription of oral vancomycin was added into the case definition, in addition to clinical diarrhea, laboratory diagnosis of CDAD, and the presence of pseudomembranous colitis, to increase statistical power and reduce exposure misclassification of the study by Suissa et al. [10]. Only the study by Suissa et al. differentiated between the types of NSAIDs used, and they found that only diclofenac use was associated with an increased risk of CDAD [10]. A previous study showed that the use of NSAIDs can promote acute diarrhea and trigger inflammatory bowel disease (IBS) or reactivate IBS [22]. Although the underlying mechanism of these effects was unclear, it was thought that they were caused by the alteration of the intestinal barrier and increasing intestinal mucosa permeability caused by oxidative phosphorylation inhibition within enterocytes in animal models [23, 24].

This review has several limitations, and, thus, our results should be interpreted with caution. First, the major limitation of our review is the small number of studies that met our inclusion criteria; only eight studies were included in the meta-analysis. Pépin et al. did not provide the number of participants with CDAD or control data [18]; thus, those patients were not included in the analysis. Additionally, all of the included studies were observational studies that may be associated with potential confounders such as patient baseline characteristics and other factors among the selected population, such as age, sex, concomitant medications, and comorbidities, which may have affected the risk of CDAD. Third, there was high heterogeneity among the studies analyzed that might be explained by the different study designs, definition of variables, or patient characteristics. Metaregression was not performed because of the small number of studies included in the analysis.

In conclusion, we found a significant association between the use of NSAIDs and having CDAD. The results of this systematic review and meta-analysis have important implications: CDAD should be considered part of the differential diagnosis when faced with patients that present with acute diarrhea and a history of recent NSAID exposure, in addition to the use of antimicrobial or acid-suppressive agents. However, it should be noted that the results of this meta-analysis of observational studies can only demonstrate the association, not the causal relationship. Further studies are necessary to evaluate whether any specific types of NSAIDs can increase the risk of CDAD or if the course of CDAD treatment should be prolonged if NSAIDs are being used concurrently.

## Supplementary Material

Funnel plot to assess publication bias of included studies. Circles on the right of vertical line had odds ratio more than 1.39. Circles on the right of vertical line had odds ratio less than 1.39.

## Figures and Tables

**Table 1:** Characteristics of included studies.

Study (year)	Design	Participants characteristics	CDAD criteria	Controls	Participants (*n*)
Dial et al. [5]	Population-based case-control studies	Participants aged ≥18 years Data were obtained from United Kingdom GPRD	Presence of a first positive *C. difficile *toxin assay and/or a clinical diagnosis recorded by general practitioner; not hospitalized in the year prior to the index date	Age matched controls were not hospitalized in the year prior to the index date	CDAD, 1,233 Controls, 12,330

El Feghaly et al. [16]	Case-control study	Children with diarrhea from inpatient, outpatient, and emergency department visits	Diarrhea with positive *C. difficile* PCR	Symptomatic children with no *C. difficile*	CDAD, 65 Symptomatic controls, 37

Loo et al. [15]	Prospective cohort study	Participants aged ≥18 years admitted to units with a historically high or low incidence of *C. difficile* infection	Presence of diarrhea and a positive *C. difficile* cytotoxin assay or toxigenic culture, presence of diarrhea and an endoscopic diagnosis of pseudomembranes, or a pathological diagnosis of *C. difficile* infection	Asymptomatic *C. difficile* colonization Neither infection nor colonization	CDAD, 117 *C. difficile* colonization, 307 Neither colonization nor infection, 3719

Manges et al. [14]	Nested case-control study	Subjects enrolled in a large cohort study supported by FRSQ *Clostridium difficile* Consortium	(1) Presence of diarrhea and laboratory confirmation of *C. difficile* with positive toxin assay results (described below), (2) acute diarrhea without an alternate explanation and diagnosis of pseudomembranes, or (3) histologic or pathologic diagnosis of pseudomembranous colitis	Matched controls in a 1 : 2 ratio according to sex, age (±5 years), and date of hospitalization (±30 days)	CDAD, 25 Controls, 50

Naggie et al. [13]	Case-control study	Participants aged ≥18 years	Diarrhea (increased stool output and unformed stool) without another etiology and a positive *C. difficile* toxin assay Onset in the community or within 72 hours of admission to a health care center	Matched controls by geographic location	CDAD, 66 Controls, 114

Pépin et al. [18]	Retrospective cohort study	Adult patients hospitalized at least once in the internal medicine, family medicine, or gastroenterology wards	(1) Diarrhea developed during the episode of care or within 60 days after last discharge and (2) either a stool specimen was found to have *C. difficile* toxin by the cytotoxicity assay or colonoscopy revealed changes typical of pseudomembranous colitis and/or histopathology supported that diagnosis	—	CDAD, 293 Non-CDAD, 5619

Regnault et al. [12]	Retrospective study	All patients hospitalized for IBD flares in the Gastroenterology Department of the Saint-Antoine IBD Center	Positive stool toxigenic culture and a positive stool cytotoxicity assay or, in cases of negative stool cytotoxicity assays, a positive toxigenic culture	—	*C. difficile* infection, 34 No infection, 449

Soes et al. 2014 [11]	Prospective matched case-control study	All patients aged ≥2 years that had a fecal sample submitted because of diarrhea or other gastrointestinal symptoms	Patients with diarrhea or other gastrointestinal symptoms and positive culture for toxigenic *C. difficile*	Matched controls by age, sex, and site for laboratory analyses of samples	CDAD, 177 Controls, 242

Suissa et al. [10]	Case-control study	Participants aged ≥18 years and have at least 2 years of records in the GPRD	First clinical diagnosis of CDAD, a first laboratory diagnosis of CDAD, or a first prescription of oral vancomycin	Matched controls by age, medical practice	CDAD, 1360 Controls, 13072

CDAD:* Clostridium difficile-*associated diarrhea; OR: odds ratio; RR: rate ratio; CI: confidence interval; GPRD: General Practice Research Database; FRSQ: Fonds de Recherché en Santé du Québec; PCR: polymerase chain reaction; NSAID: nonsteroidal anti-inflammatory drugs; IBD: irritable bowel syndrome.

**Table 2:** Summary of quality assessment.

Author	Selection (max. 4)	Comparability (max. 2)	Exposure (max. 3)
Dial et al. [5]	4	2	2
El Feghaly et al. [16]	3	0	2
Loo et al. [15]	3	2	3
Manges et al. [14]	4	2	2
Naggie et al. [13]	4	2	2
Pépin et al. [18]	2	2	2
Regnault et al. [12]	2	0	2
Soes et al. [11]	4	2	2
Suissa et al. [10]	4	2	2
