# Supplementary material for: Association between NSAIDs and Clostridium difficile-Associated Diarrhea: A Systematic Review and Meta-Analysis
Source: Can J Gastroenterol Hepatol. 2016 Mar 8;2016:7431838. doi: 10.1155/2016/7431838 (PMC4904696; doi:10.1155/2016/7431838)
Supplement: Supplementary file 1 — Funnel plot to assess publication bias of included studies. Circles on the right of vertical line had odds ratio more than 1.39. Circles on the right of vertical line had odds ratio less than 1.39. [file 7431838.f1.zip › Supplemental Data File.doc]

**Supplemental Data**

**Search strategy**

MEDLINE

1. clostridium difficile.mp. or exp Clostridium difficile/

2. Pseudomembranous colitis.mp. or exp Enterocolitis, Pseudomembranous/

3. toxic megacolon.mp. or exp Megacolon, Toxic/

4. antibiotic associated diarrhea.mp.

5. hospital acquired diarrhea.mp. [mp=title, abstract, original title, name of substance word, subject heading word, keyword heading word, protocol supplementary concept word, rare disease supplementary concept word, unique identifier]

6. nosocomial diarrhea.mp.

7. 1 or 2 or 3 or 4 or 5 or 6

8. anti inflammatory agents, non steroidal.mp. or exp Anti-Inflammatory Agents, Non-Steroidal/

9. nsaid*.mp. [mp=title, abstract, original title, name of substance word, subject heading word, keyword heading word, protocol supplementary concept word, rare disease supplementary concept word, unique identifier]

10. nonsteroid* anti-inflammator*.mp.

11. non-steroid* anti-inflammator*.mp.

12. nonsteroid* antiinflammator*.mp.

13. non-steroid* antiinflammator*.mp.

14. ibuprofen.mp. or exp Ibuprofen/

15. diclofenac.mp. or exp Diclofenac/

16. fenoprofen.mp. or exp Fenoprofen/

17. indomethacin.mp. or exp Indomethacin/

18. ketoprofen.mp. or exp Ketoprofen/

19. mefenamic acid.mp. or exp Mefenamic Acid/

20. naproxen.mp. or exp Naproxen/

21. piroxicam.mp. or exp Piroxicam/

22. sulindac.mp. or exp Sulindac/

23. exp Anti-Inflammatory Agents/ or nonsteroidal anti-inflammatory.mp.

24. non-steroidal anti-inflammatory.mp.

25. nonsteroidal antiinflammatory.mp.

26. non-steroidal antiinflammatory.mp.

27. nsaids.mp.

28. 8 or 9 or 10 or 11 or 12 or 13 or 14 or 15 or 16 or 17 or 18 or 19 or 20 or 21 or 22 or 23 or 24 or 25 or 26 or 27

29. 7 and 28

Embase

((('clostridium difficile'/exp or 'clostridium difficile' and [embase]/lim) or ('pseudomembranous colitis' and [embase]/lim) or ('c. difficile' and [embase]/lim) or ('toxic megacolon' and [embase]/lim) or ('antibiotic associated diarrhea' and [embase]/lim) or ('hospital acquired diarrhea' and [embase]/lim) or ('nosocomial diarrhea' and [embase]/lim)) and (('non steroidal anti inflammatory agent'/exp or 'non steroidal anti inflammatory agent' and [embase]/lim) or (nsaid* and [embase]/lim) or (antiinflammator* and [embase]/lim) or (ibuprofen and [embase]/lim) or (diclofenac and [embase]/lim) or (fenoprofen and [embase]/lim) or (indomethacin and [embase]/lim) or (ketoprofen and [embase]/lim) or ('mefenamic acid' and [embase]/lim) or (naproxen and [embase]/lim) or (piroxicam and [embase]/lim) or (sulindac and [embase]/lim)) and [english]/lim) and 'human'/de

CENTRAL

ID	Search	Hits

#1	MeSH descriptor: [Anti-Inflammatory Agents, Non-Steroidal] explode all trees

#2	nsaid*

#3	nonsteroid* anti-inflammator*

#4	non-steroid* anti-inflammator*

#5	nonsteroid* antiinflammator*

#6	non-steroid* antiinflammator*

#7	ibuprofen

#8	diclofenac

#9	fenoprofen

#10	indomethacin

#11	ketoprofen

#12	mefenamic acid

#13	naproxen

#14	piroxicam

#15	sulindac

#16	#1 or #2 or #3 or #4 or #5 or #6 or #7 or #8 or #9 or #10 or #11 or #12 or #13 or #14 or #15

#17	MeSH descriptor: [Clostridium difficile] explode all trees

#18	Pseudomembranous colitis

#19	c. difficile

#20	toxic megacolon

#21	antibiotic associated diarrhea

#22	hospital acquired diarrhea

#23	nosocomial diarrhea

#24	#17 or #18 or #19 or #20 or #21 or #22 or #23

#25	#16 and #24
